# Early outcomes after adopting robotic intracorporeal anastomosis for right hemicolectomy – a propensity-weighted analysis

**DOI:** 10.1007/s11701-026-03343-3

**Published:** 2026-03-30

**Authors:** Georgios Geropoulos, Syed Mohsin, Christos Athanasiou, Bibechan Thapa, Nayana Prakash, Arian Arjomandi Rad, Lillian Reza, Vanash Patel

**Affiliations:** 1 Department of Surgery and Cancer, Imperial College London, 10th Floor Queen Elizabeth Queen Mother Building, St Mary’s Hospital, London, W2 1NY UK; 2 Department of Surgery, Anaesthetics and Cancer, West Hertfordshire Teaching Hospitals NHS Trust, Watford General Hospital, Vicarage Road, Watford, WD18 0HB UK; 3 Oxford University Hospitals NHS Foundation Trust, John Radcliffe Hospital, Headley Way, Headington, Oxford, OX3 9DU UK

**Keywords:** Minimally invasive surgery, Postoperative recovery, Oncological quality, Real-world data

## Abstract

To compare early outcomes of extracorporeal anastomosis (ECA) versus robotic intracorporeal anastomosis (ICA) in right hemicolectomy within a UK setting. We performed a retrospective cohort study of all minimally invasive right hemicolectomies at our institution between January 2023 and October 2025. The primary outcome was total length of recovery, defined as in‑hospital stay plus days enrolled in a “virtual hospital”. Secondary outcomes were operative time, postoperative inflammatory response (day‑1 C‑reactive protein, CRP), lymph node harvest, resection margin status, and postoperative complications (Clavien–Dindo). To account for baseline differences between groups, we applied inverse probability of treatment weighting (IPTW) based on preoperative demographic and clinical variables. A total of 163 patients were included: 100 undergoing ECA and 63 robotic ICA. After IPTW adjustment, ICA was associated with a significantly shorter total length of stay (RR 0.76, 95% CI 0.68–0.85; *p* < 0.001) and reduced in‑hospital stay (RR 0.48, 95% CI 0.42–0.54; *p* < 0.001). ICA required longer operative time (mean + 21 min, 95% CI 5.2–36.7; *p* = 0.009) but resulted in a lower day‑1 CRP (31.6 mg/L, 95% CI − 44.2 to − 18.9; *p* < 0.001). Lymph node yield was significantly higher with ICA in cancer patients (+ 2.6 nodes, 95% CI 0.68 to 4.47; *p* = 0.008), and R0 resection rates were comparable (OR 1.71, 95% CI 0.17 to 17.24; *p* = 0.649). Overall postoperative complications were significantly reduced in the ICA group (OR 0.30, 95% CI 0.16–0.55; *p* < 0.001). Robotic intracorporeal anastomosis for right hemicolectomy was associated with faster recovery and fewer complications than extracorporeal anastomosis, despite modestly increased operative time.

**Supplementary Information:**

The online version contains supplementary material available at 10.1007/s11701-026-03343-3.

## Introduction

Right-sided colonic disease is commonly managed using minimally invasive surgical approaches, with proven benefits over open surgery, including postoperative pain, recovery, and wound morbidity. A key technical consideration in these procedures is the creation of the ileocolic anastomosis. Traditionally, this has been performed extracorporeally (ECA), requiring exteriorisation of the bowel through a small laparotomy. More recently, intracorporeal anastomosis (ICA), constructed entirely within the abdominal cavity, has gained increasing adoption [1].

ICA is associated with several potential advantages, including reduced traction on the mesentery, optimal orientation of the anastomosis, smaller extraction site incisions, and the option to use Pfannenstiel or transverse incisions, which are linked with lower rates of incisional hernia and improved cosmesis [1–4]. A large analysis of over 4,450 patients found that the traditional ECA technique was linked to more conversions to open surgery and more complications than ICA [5–7]. Other trials and reviews suggest ICA can speed up recovery, reduce the risk of ileus, and lead to fewer wound infections. Despite this, not everyone has adopted it, mainly due to concerns that it’s technically more challenging and takes longer to perform. The existing clinical evidence remains heterogeneous, and further high-quality observational analyses are needed comparing contemporary laparoscopic and robotic approaches [8–12].

At West Hertfordshire Teaching Hospitals NHS Trust (WHTH), we have a well-established robotic colorectal surgery program using systems from Cambridge Medical Robotics (since 2022) and Intuitive Surgical (since 2024). Our early results have been promising, with low complication and conversion rates, shorter hospital stays, and good cancer outcomes [13, 14]. After bringing in the da Vinci Xi system, we moved to a fully robotic program, and ICA became our standard practice for right hemicolectomies. Prior to this transition, all five surgeons were fully trained and proficient in conventional laparoscopic techniques. After completing their respective robotic surgery learning curves, they assumed supervisory roles and provided structured training to resident surgeons.

This study aims to compare the early results of these two anastomotic techniques for right hemicolectomy at our high-volume centre, focusing on recovery, cancer-related quality measures, and complications.

## Methods

### Ethics approval

This study was approved locally as a service evaluation and classified as non-research under institutional governance frameworks; therefore, formal review by a UK Health Research Authority Research Ethics Committee was not required. Data were collected retrospectively from routine clinical records and analysed in anonymised form in accordance with institutional information governance policies.

### Patient recruitment and data collection

We identified all patients who underwent elective minimally invasive right hemicolectomy between January 2023 and October 2025. We gathered clinical data from their electronic health records (Cerner Millennium), including demographics, pre-existing health conditions, operative details, and any complications. Our primary outcome was length of stay. We measured this in two ways: (i) the in-hospital stay (from the day of surgery until discharge) and (ii) the “virtual hospital” stay (the time spent being monitored at home through our structured remote program). This virtual hospital pathway, which we started in November 2023, allows for early, supported discharge. After its introduction in November 2023, the virtual hospital pathway was available to eligible patients in both the ECA and ICA groups. Patients reported their symptoms and vital signs daily, and a specialist team provided support, escalating to a phone call, clinic visit, or readmission if needed. Secondary outcomes included the length of the operation (skin incision to closure), the body’s inflammatory response (measured by C-reactive protein on day 1), cancer-related outcomes (such as lymph nodes removed and resection margin status), and postoperative complications, which we graded using the Clavien–Dindo classification.

### Statistical analysis

We summarised continuous data as medians with interquartile ranges (IQR) and compared the groups using the Mann–Whitney U test. We presented categorical data as counts and percentages, using Fisher’s exact test or the chi-square test for comparisons. To account for the fact that this wasn’t a randomised study, we used Inverse Probability of Treatment Weighting (IPTW). This statistical method helps balance the two groups by adjusting for differences in patient characteristics including age, BMI, ASA score, sex, and diagnosis, reducing potential selection bias. We calculated a propensity score for each patient and trimmed the weights to ensure stability. Our goal was to acheive the standardised mean differences for all covariates below 0.1. Since the length of stay data was skewed, our main analysis used a weighted generalised linear model. We also ran a sensitivity analysis for comparison. For other continuous outcomes (like operating time and lymph node count), we used weighted least squares regression. To account for potential chronological bias related to the introduction of the virtual hospital pathway and staged adoption of ICA, a supplementary complete-case IPTW sensitivity analysis was performed, incorporating year of surgery and primary surgeon into propensity model. In addition, a prespecified cancer-only subgroup analysis was undertaken using an IPTW model, incorporating tumour stage (TNM) alongside age, BMI, ASA grade, sex, surgical indication, and primary surgeon. We analysed complication rates and pathology data using weighted generalised linear models. For rare events where one group had zero, we used Fisher’s exact test. A p-value of less than 0.050 was considered statistically significant. All analyses were done using R and Python.

## Results

### Patient characteristics

In total, we included 163 patients: 100 had an ECA and 63 had an ICA. At baseline, the two groups were quite similar. Median [IQR] age was 72.2 [61.7–80.0] years in the ECA group and 71.0 [60.5–78.0] years in the ICA group (*p* = 0.596). A lower proportion of males underwent ICA (33%) compared with ECA (48%), but this difference wasn’t statistically significant (*p* = 0.074). Median BMI was 27.0 [23.4–31.0] kg/m² for ECA and 25.0 [23.1–28.0] kg/m² for ICA (*p* = 0.631). Median ASA grade was 2 [2–3] in both cohorts (*p* = 0.764). Median Charlson Comorbidity Index (CCI) was 5 [4–6] in both groups (*p* = 0.825), with similar estimated 10-year survival of 21 [2–53]% (*p* = 0.802). Predicted operative risk (CR-POSSUM morbidity) was 17.0 [15.0–19.2]% for ECA and 16.0 [15.0–18.0]% for ICA (*p* = 0.118), with corresponding predicted mortality of 2.6 [1.3–5.3]% and 2.6 [2.3–3.4]%, respectively (*p* = 0.631). Very few patients had chemotherapy before surgery (2% of ECA cases versus 0% in the ICA cohort (*p* = 0.520)).

### IPTW analysis

**Fig. 1:**
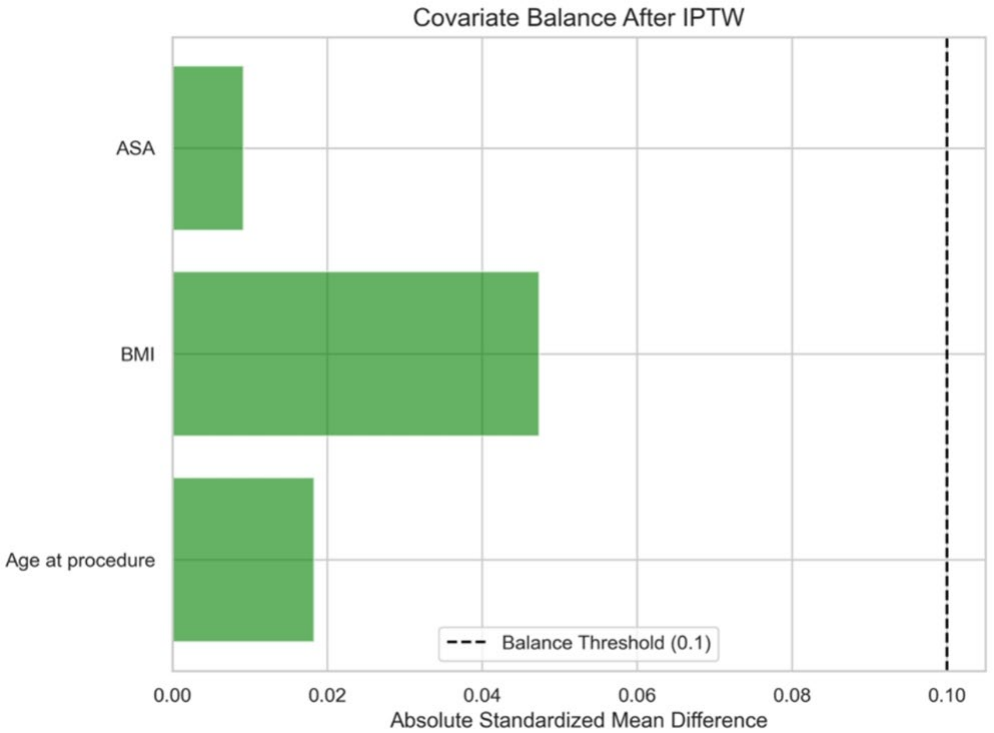
Covariate balance after IPTW. Horizontal bars show the absolute standardised mean differences (SMDs) between the ECA and ICA groups for key baseline covariates (age at procedure, BMI, and ASA grade) following IPTW

Table 1 summarises the perioperative and postoperative outcomes in the ECA and ICA groups. Figure 1 illustrates covariate balance following IPTW. IPTW analyses were performed using a complete-case approach; therefore, only patients with complete covariate and outcome data were included (*n* = 160; ECA *n* = 99; ICA *n* = 61).

**Table 1:** Unadjusted perioperative and postoperative outcomes among ECA and ICA groups (median [IQR] for continuous variables, and n (%) for categorical variables)

Outcomes	ECA	ICA
Hospital stay (days)	5 [4–6]	2 [2–3]
Operative time (mins)	163 [140–185]	178 [152–219]
CRP day 1	69 [45–95]	37 [26–59]
Lymph nodes retrieved	24 [18–30]	28 [23–34]
R1 resection	2 (2%)	1 (2%)
Ileus	11 (11%)	3 (5%)
Wound complications	5 (5%)	0 (0%)
Anastomotic leak	3 (3%)	0 (0%)
Blood transfusion	2 (2%)	0 (0%)
Unplanned ICU admission	3 (3%)	2 (3%)

*Length of stay*

**Fig. 2:**
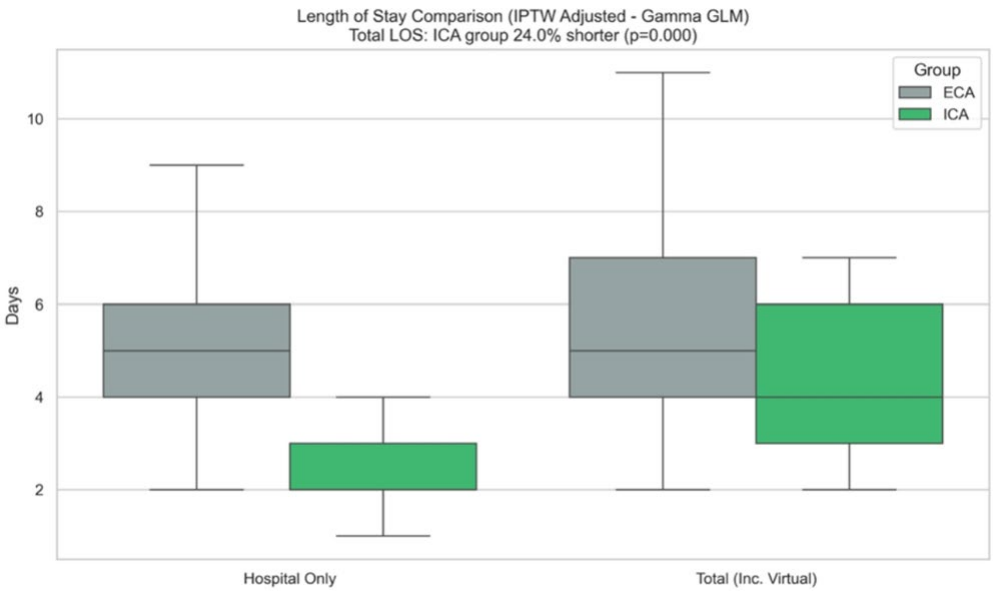
Length of stay following IPTW adjustment

The total length of stay (in-hospital plus virtual hospital) was significantly shorter for the ICA group — a 24% reduction compared to the ECA group (Rate Ratio (RR) 0.76, 95% Confidence Interval (CI) 0.68 to 0.85; *p* < 0.001). This translated to the average total stay being 1.4 days shorter for ICA patients (5.8 days for ECA vs. 4.4 days for ICA), which was consistent in sensitivity analysis using weighted least squares (95% CI − 2.16 to − 0.58; *p* = 0.001). Looking only at the time spent physically in the hospital, the benefit was even more pronounced: ICA was associated with a 53% reduction (RR 0.48; 95% CI 0.42 to 0.54; *p* < 0.001), which worked out to about 2.7 fewer days in a hospital bed (Fig. 2).

*Operative time*

**Fig. 3:**
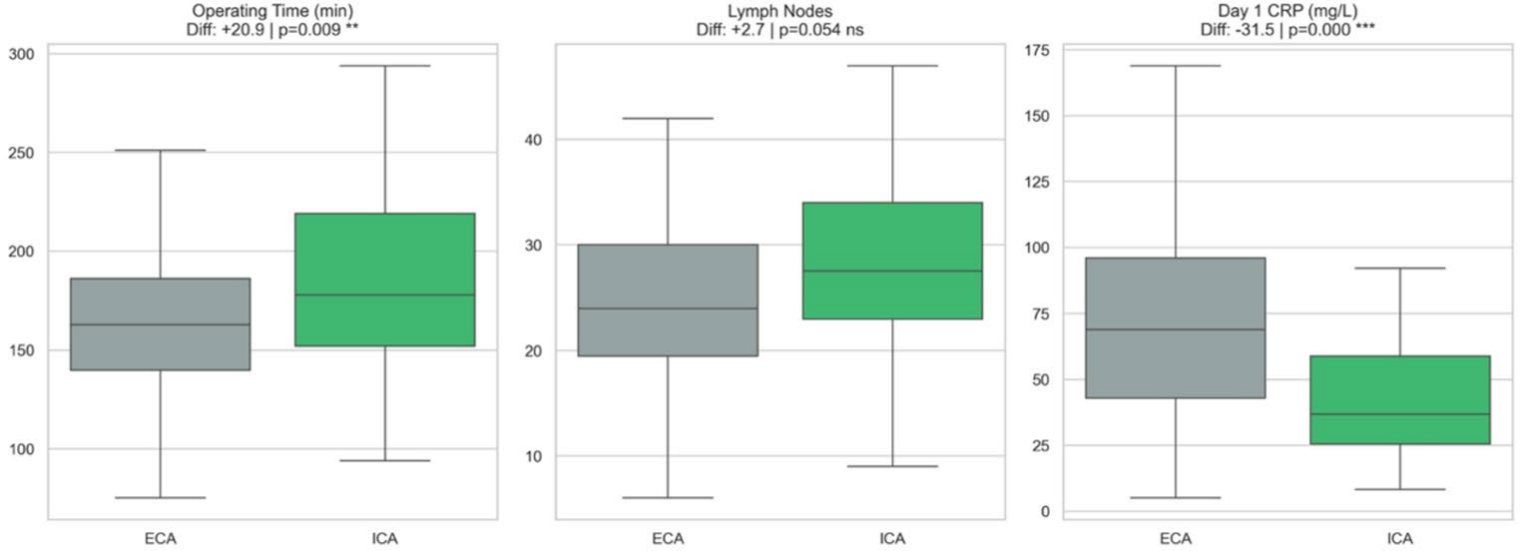
Secondary outcomes following IPTW adjustment

The ICA procedure took longer, with an adjusted mean difference of 21 min compared to ECA (95% CI 5.2 to 36.7; *p* = 0.009) (Fig. 3).

*Inflammatory response*

Patients in the ICA group had significantly lower C-reactive protein (CRP) levels the day after surgery, indicating less inflammation compared to the ECA group (adjusted mean difference − 31.6 mg/L, 95% CI -44.2 to -18.9; *p* < 0.001) (Fig. 3).

*Oncological outcomes*

**Fig. 4:**
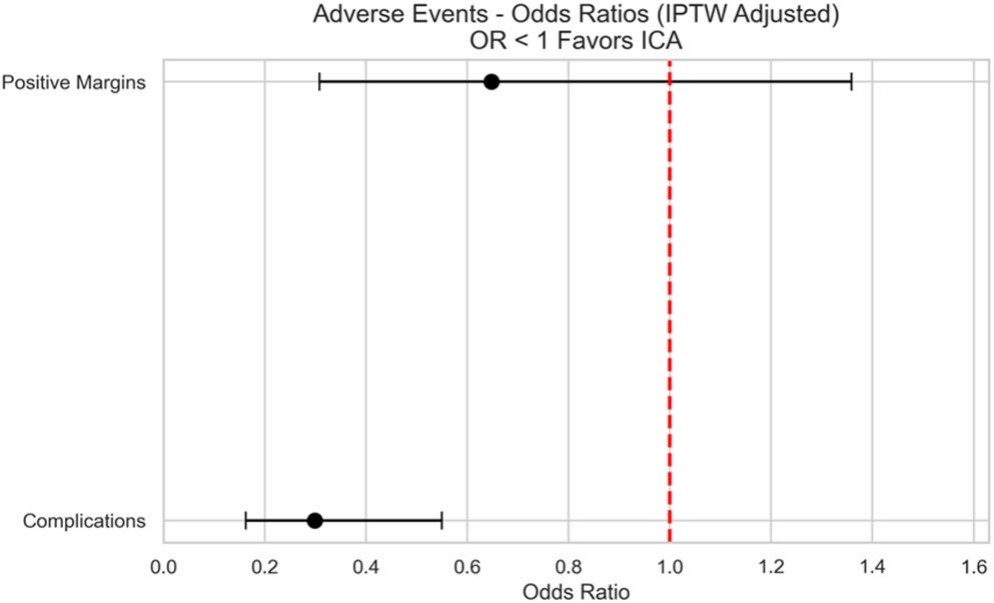
Adverse events after IPTW adjustment

The lymph node yield was slightly higher in the ICA group, with an adjusted mean difference of 2.7 more nodes removed, but this result just missed statistical significance (95% CI − 0.05 to 5.5; *p* = 0.054) (Fig. 3). The rates of clear resection margins were similar in both groups, with no significant difference found (Odds Ratio (OR) 0.65, 95% CI 0.31 to 1.36; *p* = 0.250) (Fig. 4).

*Postoperative complications*

In unadjusted analysis, there were no major complications (Grade III-V) in the ICA group, compared to 12% in the ECA group (*p* = 0.004). When examining specific issues, the ICA group had lower rates of postoperative ileus (5% vs. 11%, *p* = 0.251), wound complications (0% vs. 5%, *p* = 0.157), anastomotic leak (0% vs. 3%, *p* = 0.284) and blood transfusions (0% vs. 2%, *p* = 0.523) (Table 1). However, because these individual complications were rare, we didn’t have enough data to draw firm conclusions about each.

After weighting the data, we found that ICA was associated with significantly lower odds of having any postoperative complication (Clavien-Dindo grade ≥ I) (OR 0.30, 95% CI 0.16 to 0.55; *p* < 0.001) (Fig. 4).

*Subgroup analysis*

Covariate balance for the subgroup analyses is presented in the supplementary material (**Tables S1 and S2**).

After IPTW adjustment for age, BMI, ASA grade, sex, surgical indication, primary surgeon, and year of surgery, good covariate balance was achieved for age, ASA grade, and sex. Residual imbalance persisted for BMI and year of surgery and was considered in the interpretation of the adjusted analyses (*n* = 153; ECA *n* = 90, ICA *n* = 63).

ICA remained associated with a significantly shorter total length of stay compared with ECA (RR 0.74, 95% CI 0.63 to 0.87; *p* < 0.001), corresponding to an approximate 26% reduction in length of stay. Operative duration was longer in the ICA group, with an adjusted mean difference of 38 min compared with ECA (95% CI 20.8 to 54.9; *p* < 0.001).

After IPTW for age, BMI, ASA grade, sex, tumour stage (TNM), surgical indication, and primary surgeon, good covariate balance was achieved for age, BMI, ASA grade, sex, and TNM stage. Year of surgery was not included in this model and was therefore not balanced after weighting; this was considered in the interpretation of the adjusted analyses (*n* = 129; ECA *n* = 77, ICA *n* = 52).

ICA remained associated with a significantly shorter total length of stay compared with ECA (RR 0.65, 95% CI 0.57 to 0.73; *p* < 0.001), corresponding to an approximate 35% reduction in length of stay. Operative duration was longer in the ICA group, with an adjusted mean difference of 24 min compared with ECA (RR 1.14, 95% CI 1.07 to 1.22; *p* < 0.001). Day 1 CRP levels were significantly lower following ICA (adjusted mean difference − 36.8 mg/L, 95% CI 0.48 to 0.66; *p* < 0.001). Lymph node yield was significantly higher in the ICA group, with an adjusted mean difference of + 2.6 nodes compared with ECA (95% CI 0.68 to 4.47; *p* = 0.008). Rates of negative resection margins were comparable between groups (OR 1.71, 95% CI 0.17 to 17.24; *p* = 0.649). The overall postoperative complication rate (Clavien–Dindo grade ≥ I) was significantly lower in the ICA group (OR 0.39, 95% CI 0.21 to 0.72; *p* = 0.002).

## Discussion

In our analysis, performing a robotic ICA was linked to significantly shorter hospital stays and fewer complications compared with the traditional ECA method, even though the operation itself took longer [5, 6, 10].

The advantages of ICA are likely multifactorial. ICA may reduce tissue trauma and facilitate the construction of a tension-free, well-orientated anastomosis [1]. In addition, ICA enables specimen extraction through Pfannenstiel incisions, which reduce postoperative pain, improve cosmesis, and have low incisional hernia rates [15–17]. These technical considerations may underpin the improved recovery profile observed in our cohort and reported elsewhere, including lower wound infection rates [2, 6, 8] and reduced incisional hernia incidence [8].

Length of stay was analysed both as in-hospital stay and as total stay including virtual hospital monitoring, reflecting the introduction of a structured early supported discharge pathway during the study period. Importantly, this pathway was available to both ICA and ECA groups. However, ICA patients may have been more readily suitable for early supported discharge due to reduced wound pain and improved mobility associated with off-midline extraction sites. Virtual ward models are increasingly recognised for their ability to reduce inpatient bed utilisation without compromising safety, and reporting both in-hospital and total length of stay allowed us to distinguish between earlier discharge and overall recovery burden. The finding that ICA was associated with reductions in both metrics suggests a genuinely faster return to discharge readiness rather than simple redistribution of care to the outpatient setting.

Our findings align closely with previous reports demonstrating shorter hospital stay with ICA [1–3, 6–9]. In one study, a greater proportion of ICA patients achieved discharge within four days compared with ECA [8], while another demonstrated a median reduction of one postoperative day with ICA [18]. Furthermore, ICA has consistently been associated with lower postoperative ileus [6, 19] and reduced need for postoperative interventions (Clavien–Dindo grade III) [8], reinforcing its favourable impact on recovery trajectories.

Operative times have been shown to be comparable between ICA and ECA [2, 3, 7], whereas our own analysis and multiple large series demonstrate longer durations with ICA [8, 10, 18–21]. This trade-off between longer operative time and superior postoperative recovery appears consistent across the literature [2, 3, 7, 20]. In our cohort, major complications were observed only in the ECA group, suggesting that the increased technical complexity of ICA does not result in increased morbidity when performed within a mature minimally invasive programme.

The robotic platform may make ICA safer and more reproducible by providing surgeons with better dexterity, ergonomics, and precision. Early evidence suggests robotics might shorten the learning curve for this technique, helping more surgeons adopt it [22, 23]. In robotic right hemicolectomy, ICA has been linked to fewer conversions to open surgery, smaller incisions, and shorter hospital stays, despite the longer operating times. This all points to robotic platforms as a key tool in overcoming the technical hurdles of ICA while retaining its clinical benefits [18, 24]. A case–control study also demonstrated faster bowel recovery and fewer anastomotic and incisional complications with robotic ICA compared with laparoscopic ECA [25]. Collectively, these findings support the role of robotic platforms in lowering technical barriers to ICA while preserving its clinical benefits.

Our analysis demonstrated a significantly higher lymph node yield in cancer patients undergoing ICA compared with ECA, suggesting a potential oncological advantage with the intracorporeal approach. While several previous studies have reported no significant difference in lymph node harvest between techniques [7, 9], others have suggested a benefit with ICA [8, 9]. The increased nodal yield observed in our cohort may reflect improved mesenteric exposure, more precise vascular ligation, and reduced mesenteric traction and distortion associated with intracorporeal mobilisation and anastomosis.

This study has several limitations. As a retrospective analysis, there is potential for selection bias, and unmeasured confounders such as case complexity or perioperative management may have influenced outcomes. Although the analysis focuses on anastomotic technique, the comparison does not represent a pure technique-only assessment. The ECA group included both laparoscopic and robotic cases, whereas all ICA procedures were performed robotically. Consequently, platform-related effects, time-related era bias, and learning curve effects cannot be excluded, particularly as ICA was adopted later, following the transition to a fully robotic colorectal programme. The evolution of perioperative care pathways, including the introduction of the virtual hospital, may also have influenced length of stay outcomes. As a single-centre study, the findings may not be generalisable, and the modest sample size, particularly in the ICA group, limited power to detect uncommon complications. Previous abdominal surgery was not included in the propensity model because of insufficient data completeness, which may have contributed to residual confounding. Although propensity weighting was applied, unmeasured confounding cannot be excluded, and the findings should therefore be interpreted as real-world outcomes associated with robotic ICA rather than definitive evidence of superiority of anastomotic technique.

## Conclusion

For patients undergoing right hemicolectomy, the robotic ICA technique was associated with a shorter recovery and fewer major complications compared to ECA, despite taking longer in the operating room. We believe the benefits stem from gentler bowel handling and the use of better-tolerated non-midline incisions. While our findings support the growing trend of adopting ICA as the standard of care, larger, multicenter randomised trials are still needed to confirm these benefits.

## Supplementary Information

Below is the link to the electronic supplementary material.

Supplementary Material 1

## Data Availability

The datasets generated and analysed during the current study are not publicly available due to patient confidentiality and institutional policy but are available from the corresponding author on reasonable request.
