# Supplementary material for: Early outcomes after adopting robotic intracorporeal anastomosis for right hemicolectomy – a propensity-weighted analysis
Source: J Robot Surg. 2026 Mar 30;20(1):377. doi: 10.1007/s11701-026-03343-3 (PMC13035539; doi:10.1007/s11701-026-03343-3)
Supplement: Supplementary file 1 — Supplementary Material 1 [file 11701_2026_3343_MOESM1_ESM.docx]

# Supplementary Material

## Table S1. Covariate balance for subgroup model 1

Propensity score model including: age, BMI, ASA grade, sex, surgical indication, primary surgeon, and year of surgery.

|  | Standardised Mean Difference (Before) | Standardised Mean Difference (After) |
| --- | --- | --- |
| Age at procedure | -0.149 | 0.031 |
| BMI | -0.300 | -0.149 |
| ASA grade | -0.032 | 0.053 |
| Male sex | -0.273 | 0.091 |
| Year of surgery | 1.641 | 0.826 |

## Table S2. Covariate balance for subgroup model 2

Propensity score model including: TNM stage, age, BMI, ASA grade, sex, and primary surgeon.

|  | Standardized Mean Difference (Before) | Standardized Mean Difference (After) |
| --- | --- | --- |
| Age at procedure | -0.218 | 0.078 |
| BMI | -0.276 | 0.004 |
| ASA grade | -0.178 | 0.014 |
| Male sex | -0.235 | -0.028 |
| TNM stage | 0.141 | 0.065 |
| Consultant (surgeon) | -0.441 | -0.199 |
| Year of surgery* | 1.575 | 1.597 |

*Year of surgery was not included in the propensity score model for Subgroup Model 2.
